# Invasion, establishment, and spread of invasive mosquitoes from the *Culex coronator* complex in urban areas of Miami-Dade County, Florida

**DOI:** 10.1038/s41598-021-94202-8

**Published:** 2021-07-16

**Authors:** André B. B. Wilke, Chalmers Vasquez, Gabriel Cardenas, Augusto Carvajal, Johana Medina, William D. Petrie, John C. Beier

**Affiliations:** 1 Department of Public Health Sciences, Miller School of Medicine, University of Miami, 1120 Northwest 14th Street, Miami, FL 33136 USA; 2 Miami-Dade County Mosquito Control Division, Miami, FL USA

**Keywords:** Ecological epidemiology, Entomology, Coevolution

## Abstract

Species from the *Culex coronator* complex are Neotropical species and potential vectors of Saint Louis and West Nile viruses. *Culex coronator* was first described in Trinidad and Tobago in the early twentieth century and since then it has invaded and has been reported established in most countries of the Americas. Species from the *Culex coronator* complex were first detected in the United States in the state of Louisiana in 2004 and were subsequently detected in Florida in 2005, reaching Miami-Dade County in 2008. We hypothesize that species from the *Cx. coronator* complex are adapting to urban environments in Miami-Dade County, Florida, and are becoming more present and abundant in these areas. Therefore, our objective was to investigate the patterns of the presence and abundance of species from the *Cx. coronator* complex in the urban areas of Miami-Dade County. Here we used weekly data comprised of 32 CDC traps from 2012 to 2020 and 150 BG-Sentinel traps from 2016 to 2020. A total of 34,146 female mosquitoes from the *Cx. coronator* complex were collected, 26,138 by CDC traps and 8008 by BG-Sentinel traps. While the number of CDC traps that were positive was relatively constant at 26–30 positive traps per year, the number of positive BG-Sentinel traps varied substantially from 50 to 87 positive traps per year. Furthermore, the heat map and logistic general linear model for repeated measures analyses showed a significant increase in both the distribution and abundance of mosquitoes from the *Cx. coronator* complex*,* indicating that these species are becoming more common in anthropized habitats being able to thrive in highly urbanized areas. The increase in the distribution and abundance of species from the *Cx. coronator* complex is a major public health concern. The ability of species from the *Cx. coronator* complex to benefit from urbanization highlights the need to better understand the mechanisms of how invasive vector mosquito species are adapting and exploiting urban habitats.

## Introduction

Vector-borne diseases pose a major threat to public health^1–3^. Currently, arbovirus outbreaks are becoming more frequent worldwide. Dengue is endemic in 128 countries infecting millions of people every year^4,5^. Even though it has stabilized in recent years, the incidence of West Nile virus (WNV) is on the rise due to a a significant increase in the number of human infections, especially in North America^6–8^. Saint Louis encephalitis (SLEV) is also an epidemiologically relevant arbovirus. It can be found in most countries in the Americas ranging from Canada to Argentina. Most SLEV human cases occur in the United States, and from the 72 SLEV infections reported from 2010 to 2019, 6 resulted in death^9^.

The increase in the incidence of vector-borne diseases can be partially attributed to the increase in presence, abundance, and distribution of vector mosquito species in urban areas^10,11^. These species often have a great potential to invade and colonize new areas and greatly benefit from biotic homogenization processes and biodiversity loss caused by urbanization^12–14^. As a consequence, the increased contact between vector mosquitoes and human hosts heightens the exposure of humans to dangerous arboviruses^15^. Invasive mosquito species are among the most important vectors^16–20^. Species such as *Aedes aegypti* (L.), *Aedes albopictus* (Skuse), and *Culex quinquefasciatus* Say can rapidly adapt to and benefit from anthropogenic alterations in the environment, being able to invade and colonize urban areas^21–24^.

*Culex coronator* Dyar and Knab is a highly invasive Neotropical species^16^ that was first observed in Trinidad and Tobago in 1906^25^. It belongs to the *Culex coronator* complex with another four sibling species *Culex camposi* Dyar, *Culex ousqua* Dyar, *Culex usquatissimus* Dyar, and *Culex usquatus* Dyar^26^. These species can occur in sympatry and are only distinguishable by a few morphological features present at the larval stage and in the male genitalia^26,27^.

Species from the *Culex coronator* complex have an unknown epidemiological role in the transmission of arboviruses to humans. However, they have been found to be competent vectors of WNV^28,29^ and are considered potential vectors of SLEV^30,31^. They have greatly expanded their range and are now present in most countries of the Americas. Since its first detection in Louisiana in 2004, species from the *Cx. coronator* complex are now considered established in most of the southern states in the United States^16^. Even though the invasion of species from the *Cx. coronator* complex is well documented in the United States, the mechanisms (e.g., changes in their ecology and behavior, adaptation to local artificial aquatic habitats and resources, etc.) employed on their invasion, establishment, and colonization of new areas remain largely unknown.

Several factors could have contributed to the dispersion of species from the *Cx. coronator* complex over large geographic areas. However, their ability to thrive and establish themselves in urban areas that have undergone abrupt and substantial anthropogenic land use and land cover transformations indicates that these species are able to exploit the resources present in urban areas at the microgeographic scale (i.e., neighborhood level). Furthermore, many mosquito vector species have the potential to be passively or actively introduced into urban areas^32–34^, but only a selected few species are able to reach high abundances. It is clear that being able to exploit the resources available in urban areas at the microgeographic scale is crucial for their establishment and proliferation in urban areas^14^. Determining to what extent species from the *Cx. coronator* complex are expanding their range in urban areas at the microgeographic scale and whether they are increasing their contact with human hosts in the process is essential for the development of effective and targeted mosquito control strategies.

Species from the *Culex coronator* complex have only recently been detected in Florida with the first reports dating from 2005, reaching Miami-Dade County in 2008^35^. Since then they have been found breeding in artificial aquatic habitats such as pools, garbage cans, and fountains^36^ and have become abundant in the urban areas of the county^13^. We hypothesize that species from the *Cx. coronator* complex are adapting to urban environments in Miami-Dade County, Florida, and are becoming gradually more present and abundant in those areas. Therefore, our objective was to investigate the patterns of the presence and abundance of species from the *Cx. coronator* complex in the urban areas of Miami-Dade County.

## Results

A total of 34,146 female specimens from the *Cx. coronator* complex were collected by the Miami-Dade Mosquito Control surveillance system, 26,138 by CDC traps from July 2012 to June 2020, and 8,008 by BG-Sentinel traps from July 2016 to June 2020. From the 32 CDC traps in the surveillance system, species from the *Cx. coronator* complex were collected by a minimum of 26 traps and a maximum of 30 traps in a given year. On the other hand, from the 150 BG-Sentinel traps in the surveillance system, species from the *Cx. coronator* complex were collected by a minimum of 50 and a maximum of 87 traps in a given year (Table 1). Our results also indicate that despite having only recently been detect in Miami-Dade, species from the *Cx. coronator* complex are increasing their range and abundance in urban areas of the county, and even though they are not as abundant and widespread as *Ae. aegypti* or *Cx. quinquefasciatus*, they should be considered in future mosquito control interventions (Table 2).

**Table 1:** Species from the *Culex coronator* complex collected in Miami-Dade County, Florida from 2012 to 2020 by the CDC and BG-Sentinel traps.

Date	CDC traps		BG-sentinel traps	
	Mosquitoes collected	Positive traps	Mosquitoes collected	Positive traps
07/2012–06/2013	2176	29	N/A	N/A
07/2013–06/2014	3178	28	N/A	N/A
07/2014–06/2015	3514	27	N/A	N/A
07/2015–06/2016	3813	26	N/A	N/A
07/2016–06/2017	1017	29	1687	50
07/2017–06/2018	4439	29	3250	72
07/2018–06/2019	3270	30	2071	87
07/2019–06/2020	4731	30	1000	65

**Table 2:** Epidemiologically relevant mosquito vector species collected in the urban areas of Miami-Dade County, Florida from 2016 to 2020 by the BG-Sentinel traps.

Date	*Aedes aegypti*		*Aedes albopictus*		*Culex coronator*		*Culex nigripalpus*		*Culex quinquefasciatus*	
	Mosquitoes collected	Positive traps	Mosquitoes collected	Positive traps	Mosquitoes collected	Positive traps	Mosquitoes collected	Positive traps	Mosquitoes collected	Positive traps
07/2016–06/2017	45,720	133	177	32	1687	50	2835	79	121,606	133
07/2017–06/2018	37,004	131	289	53	3250	72	18,171	118	169,495	131
07/2018–06/2019	37,515	148	542	60	2071	87	3772	98	107,480	148
07/2019–06/2020	32,224	148	626	57	1000	65	9737	127	92,973	148

**Figure 1:**
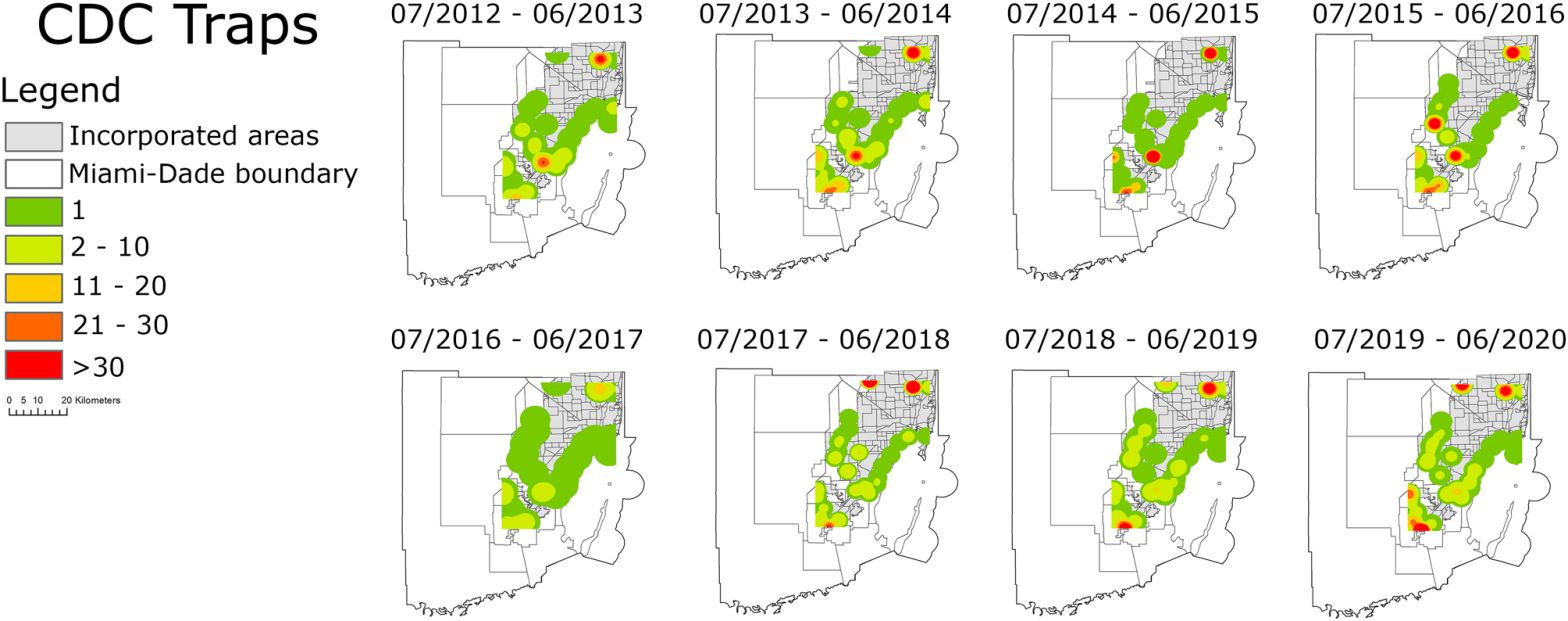
Heat map based on the relative abundance of species from the *Culex coronator* complex collected by the 32 CDC traps in Miami-Dade County, Florida from July 2012 to June 2020. The color gradient represents the sum of species from the *Cx. coronator* complex collected by each CDC trap in a 12 months period. The figure was produced using ArcGIS 10.2 (Esri, Redlands, CA), using freely available layers from the Miami-Dade County’s Open Data Hub—https://gis-mdc.opendata.arcgis.com/.

The heat map based on the Kernel density estimator of the relative abundance of species from the *Cx. coronator* complex collected by the CDC traps indicates that since their first detection in 2008, they have become abundant in the peri-urban areas of Miami-Dade County. The presence and abundance of specimens from the *Cx. coronator* complex remained constant from 2012 to 2020 indicating that these species are adapted to the conditions and resource availability found in those areas. Furthermore, specific locations with optimum conditions continually yielded high abundances of species from the *Cx. coronator* complex throughout all 8 years of data obtained by the CDC traps (Fig. 1).

**Figure 2:**
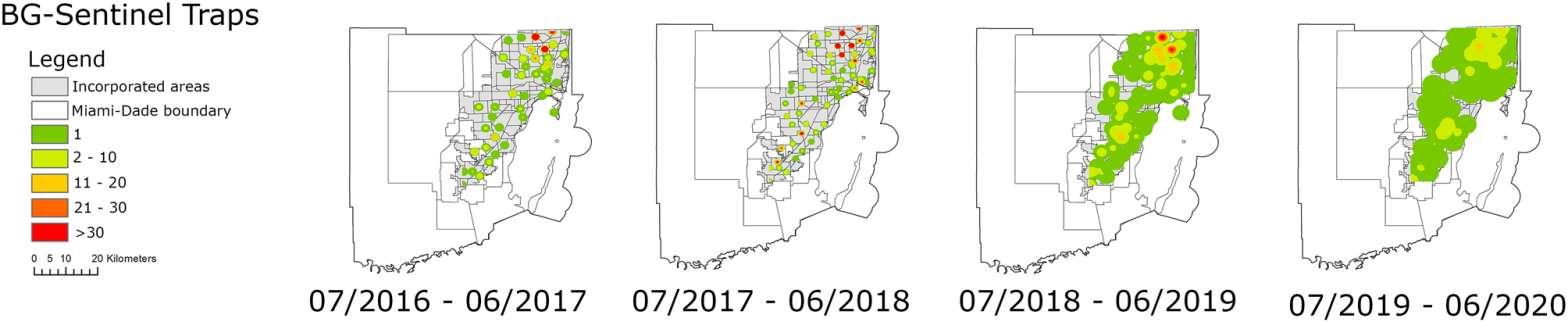
Heat map based on the relative abundance of species from the *Culex coronator* complex collected by the BG-Sentinel traps in Miami-Dade County, Florida from July 2016 to June 2020. The color gradient represents the sum of species from the *Cx. coronator* complex collected by each BG-Sentinel trap in a 12 months period. The figure was produced using ArcGIS 10.2 (Esri, Redlands, CA), using freely available layers from the Miami-Dade County’s Open Data Hub—https://gis-mdc.opendata.arcgis.com/.

The heat map based on the Kernel density estimator of the relative abundance of species from the *Cx. coronator* complex collected by the BG-Sentinel traps showed a clear increase in their presence and abundance in urban areas. Since 2016, when the surveillance system results showed that species from the *Cx. coronator* complex had limited presence and abundance relegated to specific and well-defined locations in the county, they have greatly increased their presence and abundance over the years. Our results showed a gradual increase in presence and abundance of species from the *Cx. coronator* complex in urban areas. The increase of the kernels of the heat maps indicates that more traps collected more species from the *Cx. coronator* complex in a more consistent way over the years culminating in the colonization of most of Miami-Dade urban areas by *Cx. coronator* in 2020. Furthermore, our results indicate that even though fewer mosquitoes from the *Cx. coronator* complex were collected from July 2019 to June 2020 than from July 2016 to June 2017 they were more homogeneously distributed throughout Miami-Dade. As a result, the kernel density heatmap shows larger and more widespread dots ranging from 1 to 20 mosquitoes per trap (Fig. 2).

**Figure 3:**
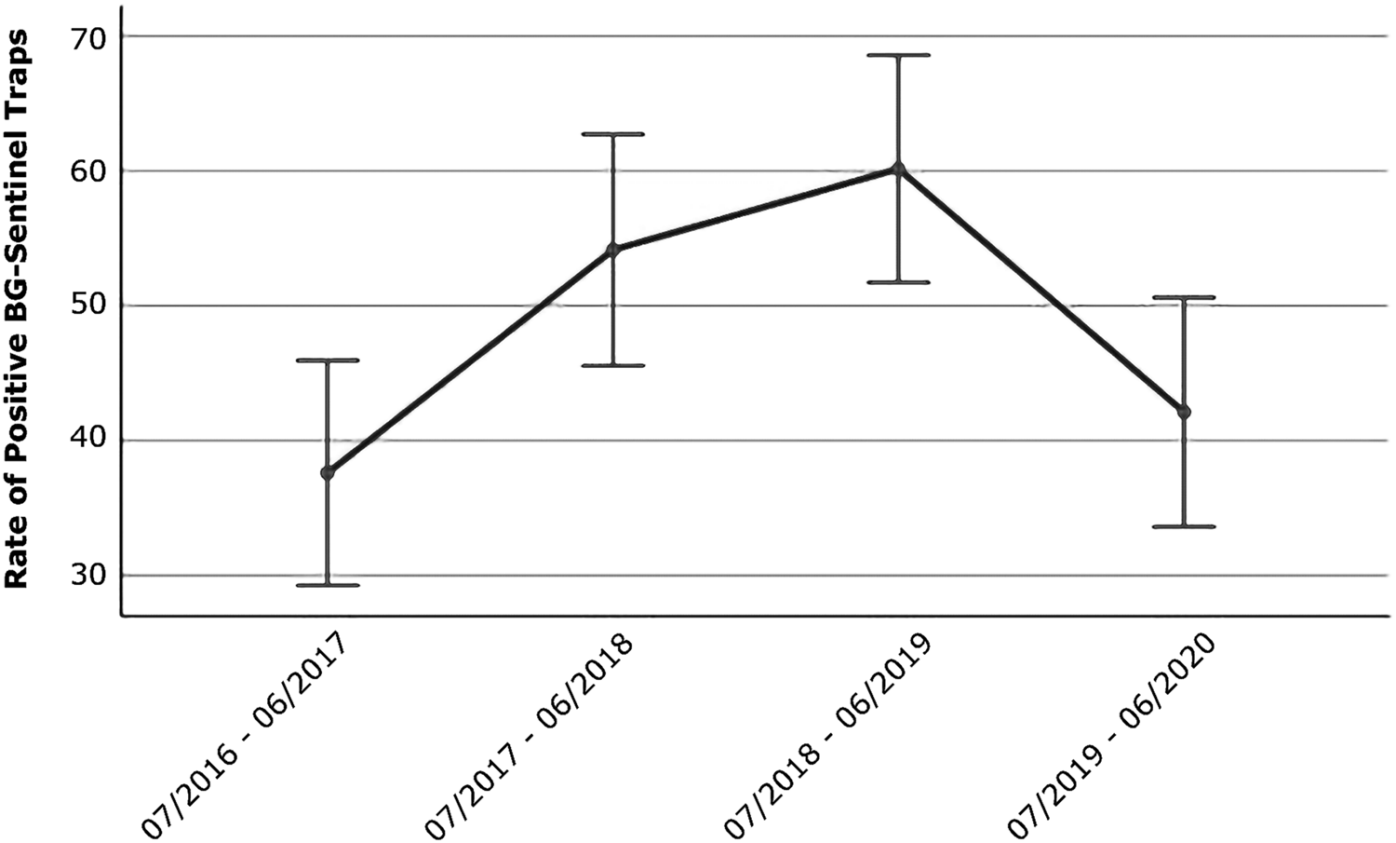
Graph of means of positive BG-Sentinel traps for species from the *Culex coronator* complex collected from July 2016 to June 2020 in Miami-Dade County, Florida. Each point displays the mean value; the whisker interval represents a 95% confidence interval standard error; the Quadratic effect was significant with a *P* value of < 0.001.

The logistic general linear repeated measures model of the mean of positive BG-Sentinel traps for species from the *Culex coronator* complex collected from July 2016 to June 2020 resulted in a significant quadratic effect over the 4 years of collections yielding a *P* value of < 0.001. The estimated 95% confidence interval standard errors did not show a linear increase but a quadratic curve, in which more BG-Sentinel traps collected significantly more mosquitoes from the *Cx. coronator* complex from July 2017 to June 2018 and July 2018 to June 2019 when compared to the number of positive traps from July 2016 to June 2017. On the other hand, even though more traps were positive for species from the *Cx. coronator* complex from July 2019 to June 2020 when compared to July 2016 to June 2017 the difference was not significant (Fig. 3).

## Discussion

Anthropogenic changes in the environment are major drivers of biotic homogenization of species, greatly benefiting species that can exploit the resources available in urban areas. Our results show that species from the *Cx. coronator* complex successfully invaded and colonized Miami-Dade County becoming one of the most abundant mosquito species with epidemiological relevance. Since its introduction in Miami-Dade County in 2008, species from the *Cx. coronator* complex gradually increased their presence and abundance. First, they increased their presence and abundance in the peri-urban areas, then they subsequently invaded and colonized the urban areas of Miami-Dade, ranging from residential areas with low population densities to highly urbanized areas comprised mostly of skyscrapers. Regardless of the availability of several species occurrence record studies, to our knowledge, this is the first study to assess the microgeographic demographic expansion of species from the *Cx. coronator* complex in urban areas in the United States.

The increase in presence and abundance of species from the *Cx. coronator* complex in Miami-Dade represents a major public health concern. One of the side effects of urbanization is the health disparities due to monetary inequities resulting in underserved populations that are more exposed to infectious diseases, including vector-borne. People living in lower-income neighborhoods are more exposed to vector mosquitoes^37^. Some houses in these under-served neighborhoods do not have air conditioning or screens on windows, and for these reasons, people tend to spend more time outdoors and are more exposed to mosquito bites during the night when species from the *Cx. coronator* complex are more active^38^. Furthermore, these areas have higher concentrations of homeless people that are constantly exposed to vector mosquito bites. The size of the homeless population fluctuates in Miami-Dade during the year, increasing substantially during the colder months of the year due to homeless people seeking warmer environments^39^. The homeless migration to Miami-Dade coincides with bird migration passing through Miami migrating south. The introduction of another mosquito vector species such as the species from the *Cx. coronator* complex that are adapted to urban environments and are recognized as an efficient vector of WNV can drastically increase the likelihood of WNV spillover from birds to humans^13,28^.

The mechanisms used by invasive species to adapt their ecology and behavior to urban environments are not fully understood. It is still unknown what allows a given species to successfully invade and colonize a new area. For example, the invasive species *Culex panocossa* Dyar has been detected in great numbers in natural areas in Miami-Dade County^40^ but has not been detected in peri-urban and urban areas^13^. The most widely accepted hypothesis is that *Cx. panocossa* close association with water lettuce plants, commonly found in natural areas in Miami-Dade but not in peri-urban and urban areas, is a limiting factor for its expansion into urban areas^41^. Furthermore, regional variation has also to be taken into consideration. For example, even though species from the *Cx. coronator* complex are considered to be invasive vector species well adapted to urban environments in the United States^16^, in Brazil they are not commonly found in urban areas^42–44^.

Even though invasion, establishment, and colonization of new areas by vector mosquito species have been well documented^16,35^, there are currently no contingency plans or specific guidelines to deter and mitigate both mosquito geographic and demographic expansions. Our results showed that since they were abundantly found in the outskirts of the urbanized areas of Miami-Dade and commonly collected by the CDC traps, species from the *Cx. coronator* complex were able to gradually colonize most of Miami-Dade incorporated areas in less than a decade, as uncovered by the results obtained by the BG-Sentinel traps, highlighting the need for the development of integrated and effective mosquito surveillance and control strategies at the national level rather than a more localized approach at the county level. Those measures together with the Integrated Vector Management (IVM) in the form of source reduction of potential artificial and natural aquatic habitats, improvement of environmental ordinance guidelines and policies, and adequate sanitary infrastructure, especially for underserved populations to attenuate social inequities are crucial for thwarting the invasion and expansion of vector mosquito species and prevent non-endemic areas from becoming endemic for arboviruses^45,46^.

The results of this study can be used to increase awareness of the danger of the introduction of invasive species. The implementation of mosquito surveillance systems capable of detecting invasive species at an early stage is paramount for the development of effective contingency plans and targeted mosquito control strategies. In this context, Miami-Dade is a gateway city and consequently has the potential to export invasive mosquito species to other parts of the contiguous United States that have suitable resources and conducive conditions to support mosquito populations.

Recently, the invasive species *Aedes vittatus* (Bigot, 1861), a primary vector of arbovirus in Europe and Asia, has been detected in Cuba and the Dominican Republic. Subsequent phylogenetic analysis indicated that *Ae. vittatus* had been introduced multiple times into the Caribbean region^47^. Due to the increased connectivity between the Caribbean region and Miami-Dade, the introduction of *Ae. vittatus* to Miami-Dade is considered unavoidable and should be monitored closely.

The continuous environmental degradation due to anthropogenic changes in the environment is a major driver for the introduction, colonization, and spread of vector mosquito species. A new and improved mosquito control framework is urgently needed to prevent both the introduction of mosquito vector species to non-endemic regions (i.e., the introduction of *Ae. vittatus* in the United States) and to manage the spread of invasive species that have already been introduced and are spreading to new areas (i.e., range expansion of species from the *Cx. coronator* complex in the United States and *Ae. albopictus* in Europe^16^).

Essential information about species from the *Cx. coronator* complex biology, behavior, and physiology is currently lacking. Most of the information currently available has been acquired through adult mosquito surveillance systems that focus on female mosquitoes. Due to caveats in the morphological identification, the correct identification of adult specimens from the *Cx. coronator* complex is only possible by observing differences in the male genitalia. For this reason, it is so far unknown what species from the *Cx. coronator* complex are present in the United States, and if one or more species from the *Cx. coronator* complex are more prone to adapt to urban environments than others and should be targeted in control strategies. The correct species identification is critical for the precise assessment of behavioral traits such as blood- and sugar-feeding, mating, resting, etc. Without that information, it is impossible to determine what environmental resources are driving the population dynamics of *Cx. coronator* and the other species in the *Cx. coronator* complex.

This study can serve as a stepping stone to future studies to determine what invasive mosquito vector species represent a risk to public health and should be targeted by mosquito control operations. Furthermore, there is no coordinated mosquito surveillance at the international and national level, which is essential to prevent the introduction and spread of invasive mosquito vector species to new areas. The introduction of invasive mosquito vector species can have an unpredictable impact on public health and represent one of the most important challenges in mosquito control and vector-borne disease transmission.

## Methods

### Study design

This study used a longitudinal design to assess the presence and relative abundance of species from the *Cx. coronator* complex in peri-urban and urban areas in Miami-Dade County, Florida. Miami-Dade is the most populous county in Florida, with approximately 3 million people. Current predictions indicate that Miami-Dade will gain 700,000 more residents by 2030^48^ causing a substantial increase in urbanization processes. Miami-Dade has a year-round wet sub-tropical climate that is conducive for the year-round proliferation of mosquitoes^13^. Miami-Dade is also a destination for many bird species that are migrating south seeking warmer weather conditions^49^. All those factors together make Miami-Dade a hotspot for WNV transmission. In 2020 alone, 59 locally transmitted human cases of WNV were reported in Miami-Dade by the Florida Department of Health^50^.

### Mosquito collection

**Figure 4:**
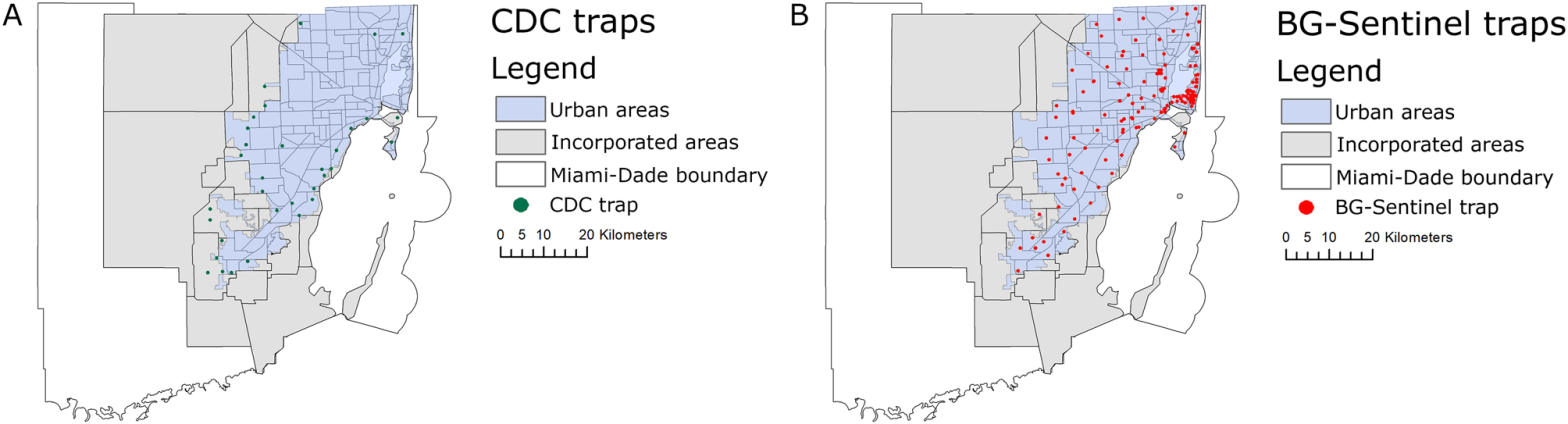
Map of Miami-Dade County, Florida. (**A**) Location of the 32 CDC traps; and (**B**) location of the 150 BG-Sentinel traps. The figure was produced using ArcGIS 10.2 (Esri, Redlands, CA), using freely available layers from the Miami-Dade County’s Open Data Hub—https://gis-mdc.opendata.arcgis.com/.

We used data obtained by BG-Sentinel (Biogents AG, Regensburg, Germany), and CDC traps. CDC traps were located in natural areas in the outskirts of the incorporated urban areas of Miami-Dade County, whereas BG-Sentinel traps were located in urban areas^13^. A total of 133 BG-Sentinel traps were deployed from July 2016 to June 2018. In July 2018, 17 new BG-Sentinel traps were added totaling 150 BG-Sentinel traps that were used from July 2018 to June 2020. A total of 32 CDC traps were deployed from July 2012 to June 2020 (Fig. 4)^13^. All traps were deployed weekly for 24 h and baited with CO_2_ using a container filled with 1 kg of dry ice pellets^13,51^. All collected mosquitoes were transported to the Miami-Dade County Mosquito Control Laboratory and morphologically identified to species using taxonomic keys^52^. Since both the BG-Sentinel and the CDC trap mainly attract females seeking hosts male mosquitoes were not included in the analysis.

### Geospatial analysis

We used a Kernel density estimator to calculate the magnitude-per-unit area (number of input per point (i.e., species from the *Cx. coronator* complex relative abundance)) from both the BG-Sentinel and CDC traps using ArcGIS 10.2 (Esri, Redlands, CA)^53^. We used the geodesic method for densities in square kilometers with an output cell size of 0.001. We used freely available layers from the Miami-Dade County’s Open Data Hub—https://gis-mdc.opendata.arcgis.com/. We used a logistic general linear model (GLM) for repeated measures for the number of BG-Sentinel traps that collected mosquitoes from the *Cx. coronator* complex on each year (positive traps) as the dependent variable, with time as the independent variable. Since this study posed less than minimal risk to participants and did not involve endangered or protected species the Institutional Review Board at the University of Miami determined that the study be exempt from institutional review board assessment (IRB Protocol Number: 20161212).
